# Performance Evaluation of Cooperative OMA and NOMA Systems in 6G Deployment Scenarios

**DOI:** 10.3390/s22113986

**Published:** 2022-05-24

**Authors:** Preksha Jain, Akhil Gupta, Neeraj Kumar, Gyanendra Prasad Joshi, Woong Cho

**Affiliations:** 1 School of Electronics and Electrical Engineering, Lovely Professional University, Phagwara 144001, Punjab, India; prekshajain248@gmail.com (P.J.); akhilgupta112001@gmail.com (A.G.); 2 Department of Computer Science and Engineering, Thapar Institute of Engineering & Technology, Patiala 147004, Punjab, India; neeraj.kumar@thapar.edu; 3 School of Computer Science, University of Petroleum and Energy Studies, Dehradun 248007, Uttarakhand, India; 4 Department of Computer Science and Engineering, Sejong University, Seoul 05006, Korea; joshi@sejong.ac.kr; 5 Department of Software Convergence, Daegu Catholic University, Gyeongsan 38430, Korea

**Keywords:** NOMA, OMA, optimization, system energy efficiency

## Abstract

Optimization of the energy efficiency, fairness, and rates of the system is a vital part of communication systems. Multiple access techniques have a huge potential to enhance such performance parameters. This paper studies the performance of NOMA and OMA systems in a singular cell environment, where the cellular users are distributed randomly, and cooperative relays are considered for better system reliability. The relay nodes forward the signals to the cell-edge users. This paper considers a practical scenario where all the relay equipment is distributed with non-uniform battery power levels. The performance of OMA and NOMA schemes is compared based on the key performance indicators: sum rate, fairness, and energy efficiency. The fairness factor determines fairness in the allocation of resources to all the system’s users. The performance of the two schemes is assessed in three deployment scenarios: urban, suburban, and rural scenarios. Through numerical results, it is proved that the performance of the NOMA dominates the OMA scheme.

## 1. Introduction

Over the last decade, revolutionary growth in wireless communication networks has been seen. The fifth-generation (5G) wireless communication systems back numerous rising verticals, including machine-to-machine (M2M) communications, enhanced mobile broadband, and applications such as high-speed entertainment and multimedia, virtual and augmented reality (VAR), etc. However, the 5th generation wireless mobile communication network may not be fully capable of meeting the up-surging demands of data traffic, spectral efficiency, massive connectivity, and capacity while providing fairness amongst the users. Therefore, researchers have shifted their interest in developing the 6G of the wireless communication network. 6G explores the THz communication band, which ranges from 0.1 THz to 10 THz. Researchers are analyzing communication in the THz band, as it has huge unexplored bandwidth, and it is expected to provide an edge over the bequest networks. Moreover, the short wavelengths of the THz wavelet huge spatial multiplexing deliver an amazingly exact output in sensing, spectroscopy, imaging, and numerous more 6G applications. Further, multiple access techniques can play a huge role in fulfilling such demands. To increase spectral efficiency, the non-orthogonal multiple access (NOMA) technique has attracted considerable attention [1,2,3]. The conventional orthogonal multiple access (OMA) techniques allocate orthogonal resources exclusively to a single user. Therefore, OMA does not hold for the required spectral efficiency to sustain 6G requirements. Time-division multiple access (TDMA), frequency division multiple access (FDMA), and code division multiple access (CDMA) are examples of the OMA scheme. NOMA is of two types—power domain and code domain [4]. NOMA uses the same resource block to serve multiple users; therefore, it utilizes the spectrum efficiently, providing better connectivity than the conventional OMA schemes.

In contrast with OMA, NOMA does not divide the available bandwidth amongst users and allocates a single frequency channel to multiple users. This results in higher system throughput, as the whole spectrum is available for each user for transmission. We focus on the power domain NOMA and refer to the power domain NOMA as NOMA for notation simplicity.

Superposition coding and successive interference cancellation (SIC) are two main techniques used in NOMA. The main advantage of NOMA is that it can serve multiple users simultaneously from a single source by exploiting superposition coding, where a power coefficient is allocated to each user, and the transmitter superimposes the signals through the linear addition of the signals of multiple users [5]. At the receiver, all the signals are ordered according to the received signal strength. First, the signal with the highest strength is decoded and is deducted from the superimposed signal received. By repeating this action consecutively, the user extracts the intended signal. The weaker signals are eliminated from the residue. This process is called SIC, and it can cancel only those interferences whose channel conditions are known at the transmitter.

However, NOMA also faces many challenges. The foremost is due to the hardware, processing, and SIC computation complexity. Moreover, the modulation implementations are nontrivial in adaptive coding and time-varying interference scenarios. The associated error in coding, propagation and decoding declines the whole system’s performance. For example, suppose there is a decoding error in the signals of previous users. In that case, this error gets carried forward to the remaining users. In conventional OMA, the users with poor channel conditions receive poor quality signals and may temporarily be suspended from service [6,7]. NOMA allocates power depending on users’ fairness, according to the users’ requirements or Quality of Service (QoS). In NOMA, the users are ordered in accordance with their channel strength. Hence, it overcomes the problem of fairness by ordering users according to and allocating more power to users with bad channel quality. Fairness amongst the users is maintained with intelligent power allocation assignments used in NOMA. To guarantee QoS, the users are ordered in accordance with their QoS requirements, and the power is allotted accordingly.

Available orthogonal resources are limited; therefore, non-orthogonal resources have proficiency in extending the number of simultaneous connections. Hence, NOMA can provide massive connectivity. The traditional OMA systems depend on access-grant requests, and users have to make scheduling requests to the base station (BS); in response, the BS sends the clear-to-send signal. This leads to high transmission latency. Scheduling is not required in NOMA, resulting in low transmission latency [8]. In a cell system, the users located at the cell edge suffer from poor channel quality signals and may not be able to form a direct communication link with the BS. The cooperation of relay nodes in NOMA helps in increasing the reception reliability and spectral efficiency of the cell edge users [9]. Saving energy of the system is one of the important considerations in designing the 5G wireless communication systems. In cooperative NOMA, the BS sends signals intended for the edge-users to the relays located comparatively nearer to the BS. Hence, in cooperative NOMA, signals can reach the edge users by consuming the condensed transmission power of the BS. Thus, it helps in moving towards greener communication. An increase in the system’s coverage area is also aided by cooperative NOMA.

## 2. Materials and Methods

### 2.1. Background of Research

NOMA was studied in various cooperative relaying systems [3,10,11,12]. In [10], spectral efficiency was improved by proposing a cooperative relaying in NOMA. The authors of [11] showed that NOMA in coordinated and direct relay transmission is superior to NOMA in non-coordinated and direct relay transmission in terms of sum capacity and outage probability. Global energy efficiency maximization in cooperative NOMA by exploring optimal power allocation schemes was investigated in [12]. Outage probability and outage capacity of cooperative NOMA were studied in [3] by exploiting the fact that the information of messages of other users is present in advance with users having good channel conditions.

In [7], the authors compared uplink OMA and NOMA from a fairness perspective. Based on the maximum likelihood receiver, the authors of [13,14] proposed a resource allocation scheme for NOMA. A comparison of OMA and NOMA in terms of spectral efficiency on the uplink channel was studied in [15]. In [16], a comparison of NOMA and OMA was carried out for a simple two-user multiple-input multiple-output (MIMO) system in terms of sum rate. In [17], for a single-input single-output (SISO) system, the superiority of NOMA over OMA was shown with equal power allocation coefficients and degree of freedom (DoF), whereas [4] showed rate superiority of MIMO-NOMA over MIMO-OMA considering arbitrary power coefficients and equal degree of freedom (DoF). In [18,19], a comparative analysis of the capacities of MIMO-NOMA and MIMO-OMA is studied.

Dynamic power allocation was studied in [20] for optimizing energy efficiency in the NOMA system. In [21], with partial channel information of the NOMA environment, the performance of a multi-user scenario is studied in terms of sum rate and outage probability and compared with the OMA system for a two-user case. In [22], a scheme was designed to select one near and far user and switch between OMA and NOMA for performance gain and study of outage probability of distant users. The resource allocation problem is solved for OMA and NOMA in [23]. In [24], a comparative analysis between OMA and NOMA schemes in a finite block-length regime was carried out to investigate the performance of the total link-layer rate. In [25], a joint bandwidth control scheme was studied, incorporating both NOMA and OMA techniques into a unified scheme. The comparison analysis of this paper with existing works is presented in Table 1.

### 2.2. Contributions

Conforming to the survey of the literature, the performance of NOMA has not been thoroughly evaluated in different multi-user 6G wireless communication deployment scenarios. Therefore, we have analyzed the performance of NOMA technology using the THz channel in 6G urban, suburban and rural deployment scenarios. The users in the urban scenario are densely populated in a small space. The users in the rural area are sparsely distributed in a comparatively larger area. The users are semi-sparse in the suburban scenario in a comparatively moderate area.

Further, the performance characterization of OMA and NOMA, considering different powers of user devices, in a 6G system using the THz channel is missing in the literature.

In previous works [26] we have proposed an adaptive NOMA scheme by considering different power levels of relay equipment employing the radio frequency (RF) channel in 5G scenario.

In this paper, we consider different power levels of user equipment and study the performance comparison of the OMA and NOMA systems in terms of different key performance parameters, such as achievable rates, energy efficiency, and fairness, presented for 6G urban, suburban and rural deployment scenarios. The simulation depicts the NOMA scheme as superior to the OMA scheme in terms of key performance parameters, including average energy efficiency, fairness factor, and average sum rate.

This paper is organized as follows: In Section 2, the design of the considered system is described. Section 3 presents the rate, fairness, and energy efficiency analysis of the considered system. The concluded results are finally shown in Section 4.

Section 4 demonstrates the results of simulations. Section 5 concludes the paper. Table 2 presents the numerous notations used in the paper.

## 3. System Design

**Figure 1:**
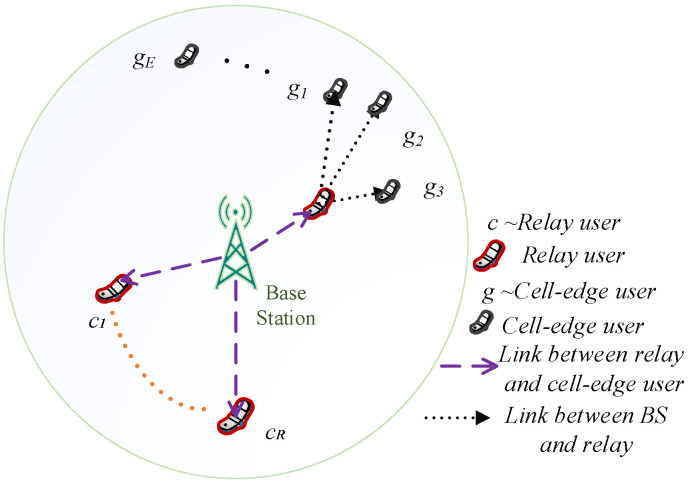
System Model.

Consider a downlink scenario employing cooperative NOMA in a cell system, with  N randomly scattered users, with one common BS. Figure 1 shows the scenario of the single-cell.

The system has  R relays and  E cell-edge users. The set of R relays is denoted as Ƈ = {$c_{1},c_{2},\ldots ,c_{R}$}, and the E cell-edge users set is represented as Ğ = {$g_{1},g_{2},\ldots ,g_{E}$}. It is presumed that each user equipment (UE) has a different battery level modeling for the practical scenario. The battery of the $\;n^{\mathrm{th}}$ relay, $c_{n,}$ where $\;c_{n}\in Ƈ$, is denoted as $\;{Ƥ}_{n}$. A Rayleigh fading scenario is considered where the BS superimposed the signals of the  M cell-edge user or sends it to $\;c_{n}$. In the cooperative NOMA system, the BS and the relay are assumed to have achieved absolute channel state information (CSI At a time, $c_{n}$ forwards the signal to maximum M cell-edge users, and its set is denoted by ${Ğ}_{M}\;$= {$g_{1},g_{2},\ldots ,g_{M}$}, such that ${Ğ}_{M}\subset Ğ$ and $\;{Ğ}_{M}”E$).

The signal transmitted by the BS for M cell-edge users to $\;c_{n}$ is given as:

(1) $${ư}_{n}=\sum_{m=1}^{M}\sqrt{a_{m}ƥ}{ѵ}_{m}\;,$$

where ${ѵ}_{m}$ denotes the modulated symbol for the $m^{\mathrm{th}}\;$ cell-edge user. Here,$\;a_{m}ƥ\;$ denotes the signal allocated power to the respective cell-edge user, such that $\;\overset{M}{\underset{m=1}{\sum }}a_{m}ƥ\leq {ƥ}_{max}$, where the maximum transmission power of the BS for $\;c_{n}$ is denoted by $\;{ƥ}_{max}$, and the power allocation coefficient is denoted by $a_{m}$, which is defined in set $\;Ά=\left\{a_{1},a_{2,}\ldots ,a_{M}\right\}$.

The signal received at $c_{n}$ is given as:

(2) $${Ϯ}_{n}=h_{b,n}{ư}_{n}+n_{n},$$

i.e.,

(3) $${Ϯ}_{n}=h_{b,n}\sum_{m=1}^{M}\sqrt{a_{m}ƥ}{ѵ}_{m}+n_{n}.\;$$

Here, the channel between BS and $\;c_{n}$ is signified by $h_{b,n}$. The $n_{n}\;$~ *CN* (0,$\;\sigma^{2}$) represents the complex additive white Gaussian noise (AWGN) vector with mean zero and variance $\sigma^{2}$ in the BS-relay link. The ${\left|h_{b,n}\right|}^{2}$ represents the gain of the THz channel [27] between $c_{n}$, and BS is represented as:

(4) $${ℎ}_{b,n}=\sqrt{\frac{1}{L}}Ɵ€(\omega ),$$

where *L* is the path loss of the THz signal. Ɵ and €(ω) respectively are antenna gain and array steering vector. From [27] €(ω) = ${\left[1,\ldots ,e^{j\pi \left[nsin\omega \right]},\ldots ,e^{j\pi \left[\left(N_{S}-1\right)\sin\omega \right]}\right]}^{}$. After receiving the signal from BS, $c_{n}\;$ forwards the superimposed signal to M cell-edge users, and it is given as:

(5) $${З}_{m}=\sum_{m=1}^{M}\sqrt{w_{m}{Ҏ}_{n}}{ѵ}_{m}.\;$$

Here, the respective cell-edge user is allocated a power coefficient depicted as $\;w_{m}{Ҏ}_{n}$, with constraint $\overset{M}{\underset{m=1}{\sum }}w_{m}{Ҏ}_{n}\leq {Ҏ}_{n\left(max\right)}.$ The maximum power of $c_{n}$ is denoted as ${Ҏ}_{n\left(max\right)}.$ The power allocation coefficient, allotted by $c_{n}\;$ for M cell-edge users is defined in set $\;w=\left\{w_{1},w_{2,}\ldots ,w_{M}\right\}$.

The signal received at $m^{\mathrm{th}}\;$ -edge user is given as:

(6) $${Ϣ}_{m}=h_{n,m}{З}_{m}+n_{m},\;$$

i.e.,

(7) $${Ϣ}_{m}=\;h_{n,m}\sum_{m=1}^{M}\sqrt{w_{m}{Ҏ}_{n}}{ѵ}_{m}+n_{m},\;$$

where the channel and AWGN are represented by $h_{m,k}$ and $n_{m}$~*CN* (0,$\;\sigma_{m}{}^{2}$)), AWGN with zero mean and variance $\;\sigma_{m}{}^{2}$, respectively, in the link between $c_{n}$ and $\;g_{m}$. The channel gain between $c_{n}$ and $g_{m}$ is denoted as $\;{\left|h_{n,m}\right|}^{2}$; the $g_{m}\;$ users can decode the message by employing SIC.

Therefore, the signal-to-interference noise-ratio (SINR) at $g_{m}$, in a coordinated relay transmission, is given as:

(8) $$SINR_{n,m}=\frac{w_{m}{Ҏ}_{n}{\left|h_{n,m}\right|}^{2}}{I_{n,m}^{1}+I_{n,m}^{2}+\sigma_{m}{}^{2}}$$

(9) $$I_{n,m}^{1}=\sum_{l\epsilon \{g_{l}|h_{n,m}<h_{n,l}\}}w_{l}{Ҏ}_{n}{\left|h_{n,m}\right|}^{2}$$

(10) $$\;\;I_{n,m}^{2}=\sum_{\begin{array}{c} k\epsilon Ƈ\\ n\neq k\;\\ \left\{{Ҏ}_{n}{\left|h_{n,m}\right|}^{2}<{Ҏ}_{k}{\left|h_{k,m}\right|}^{2}\right\} \end{array}}{Ҏ}_{k}{\left|h_{k,m}\right|}^{2}.$$

Here, due to the superimposed signal of the users with weak channel gains, the inter-NOMA interference is represented as $\;I_{n,m}^{1}$. If $c_{k}$ is transmitting the signal to $g_{m}$, in the same time slot as $\;c_{n}$, then $I_{n,m}^{2}$ is the interference encountered by $g_{m}$ due to the signals with higher product factors of the channel gain and the relay power, than its own. Therefore, $g_{m}$ encounters interference from $c_{k}$ if $\;{Ҏ}_{n}{\left|h_{n,m}\right|}^{2}<{Ҏ}_{k}{\left|h_{k,m}\right|}^{2}$, ∀t∈Ğ and  ∀k∈Ƈ.

## 4. System Analysis

The computation of signal-to-interference noise-ratio (SINR) at $g_{m}$ is described in Equation (8); the computation sum rate and fairness factor are described in this section. After that, energy efficiency has also been elaborated, and the problem of energy efficiency is formulated.

### 4.1. Sum Rate

The achievable data rate [27] at the receiver of $g_{m}$ is defined based on SINR as:

(11) $${Ř}_{n,m}=log_{2}\left(1+SINR_{n,m}\right)\;\mathrm{bps}/\mathrm{Hz}.$$

Based on the data rate, the sum rate for M cell-edge users is computed as:

(12) $${Š}_{n,m}=\sum_{m=1}^{M}{Ř}_{n,m}\;.$$

### 4.2. Fairness Factor

In a multi-user scenario, where multiple cell-edge users demand resources in the same relay, in such a case, the resources are allocated to the user’s good channel gains. Therefore, cell-edge users with poor channel gains cannot achieve desirable data rates. Hence, the QoS of the system gets degraded. The fairness factor is an important parameter in determining whether the resources are allocated efficiently to all the users. Unlike OMA, NOMA ensures fairness amongst the users with its power allocation strategies. The fairness factor [28] for N cell-edge users described by Jains’ Fairness, is given as:

(13) $${Ƒ}_{N}=\frac{{\left(\sum_{m=1}^{N}{Ř}_{n,m}\right)}^{2}}{N*\sum_{m=1}^{N}{\left({Ř}_{n,m}\right)}^{2}}\;.$$

The $\;{Ƒ}_{N}\in \left[\frac{1}{N},1\right]$, where $\frac{1}{N}$ denotes the least fairness, and 1 denotes the maximum fairness. The high fairness factor implies that the users receive identical services, which is a crucial requirement for good QoS in communication networks.

### 4.3. Energy Efficiency

Energy efficiency optimization is an important goal of the 5G communication system. The calculation of the energy efficiency of the cooperative NOMA [26,27] system is given in terms of data rate and the total power consumption for achieving the data rate. The energy efficiency achieved after the signal is received at $\;g_{m}$ and is given as:

(14) $${Ȅ}_{m}=\frac{{Ŕ}_{n,m}\;}{P_{n,m}}$$

where, $\;P_{n,m}=w_{m}{Ҏ}_{n}$, denotes the power utilization of $\;c_{n}$, for sending the message to $\;g_{m}$. The interference of the signal for $\;g_{m}$ at the relay will be due to the superimposed signals. The achieved system’s energy efficiency after M cell-edge users receives the signal is given as

(15) $$E_{T}=\frac{{Š}_{n,m}\;}{\sum_{m=1}^{M}P_{n,m}}\;.$$

## 5. Performance Analysis

The simulations are carried out to evaluate the performance of OMA and NOMA schemes in a multi-user scenario. The system is analyzed in three deployment scenarios, which are urban, suburban, and rural scenarios. The NOMA system deployed in the urban, suburban, and rural scenarios is labeled as U-NOMA, S-NOMA, and R-NOMA, respectively. The OMA system deployed in urban, suburban, and rural scenarios is labeled as U-OMA, S-OMA, and R-OMA. The numerical parameters used for implementing the scenario are depicted in Table 3.

The numerical results compare the two schemes in terms of average sum rate, average fairness factor, and average energy efficiency. A single-cell, BS-centered system is considered in three deployment scenarios; urban, suburban, and rural. The Z users in the considered system are taken from 150 to 450. All the users are randomly distributed in the cell, generating a variable count of both edge-users and relays. At maximum, respective relays simultaneously transmit the signal to  M cell-edge users.

**Figure 2:**
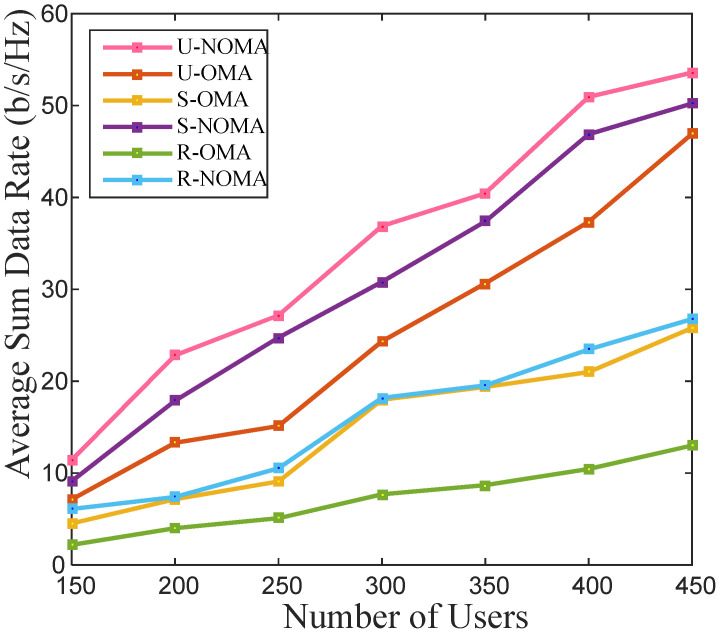
Average sum rate for a different number of total users in the cell.

The data rate achieved at $g_{m}$ in the NOMA system is given as $\;log_{2}\left(1+\frac{w_{m}{Ҏ}_{n}{\left|h_{n,m}\right|}^{2}}{\;\sum_{l\epsilon \{g_{l}|h_{n,m}<h_{n,l}\}}w_{l}{Ҏ}_{n}{\left|h_{n,m}\right|}^{2}+\sum_{\begin{array}{c} k\epsilon Ƈ\\ n\neq k\;\\ \left\{{Ҏ}_{n}{\left|h_{n,m}\right|}^{2}<{Ҏ}_{k}{\left|h_{k,m}\right|}^{2}\right\} \end{array}}{Ҏ}_{k}{\left|h_{k,m}\right|}^{2}+\sigma_{m}{}^{2}}\right)$. The data rate achieved at $g_{m}$ in the OMA system is given as $\;log_{2}\left(1+\frac{{Ҏ}_{n}{\left|h_{n,m}\right|}^{2}}{\sum_{{Ҏ}_{n}{\left|h_{n,m}\right|}^{2}<{Ҏ}_{k}{\left|h_{k,m}\right|}^{2}}{Ҏ}_{k}{\left|h_{k,m}\;\right|}^{2}+{\left|h_{b,m}\;\right|}^{2}{Ҏ}_{b}+\sigma_{m}{}^{2}}\right)$, where the channel gain of the link between BS and $\;g_{m}$ is represented by ${\left|h_{b,m}\;\right|}^{2}$, and ${Ҏ}_{b}$ is the transmitted BS power for $\;g_{m}$. The OMA achieves more interference than NOMA; hence, the achievable capacity in the case of OMA is less than NOMA. With a varying total number of users in the cell, Figure 2 presents an average sum rate analysis of cell-edge users in different deployment scenarios.

In contrast to OMA, NOMA can simultaneously transmit the signal to multiple users; therefore, the achievable average sum rate of NOMA is better than OMA. As the number of users increases, the average sum-rate upsurges. As the distance between the source and destination increases, the channel gain becomes poorer. In suburban and rural scenarios, the interference decreases but the channel gain becomes poorer as compared to the urban scenario. Therefore, the performance of each scheme is better in urban and sub-urban scenarios than in rural scenarios.

**Figure 3:**
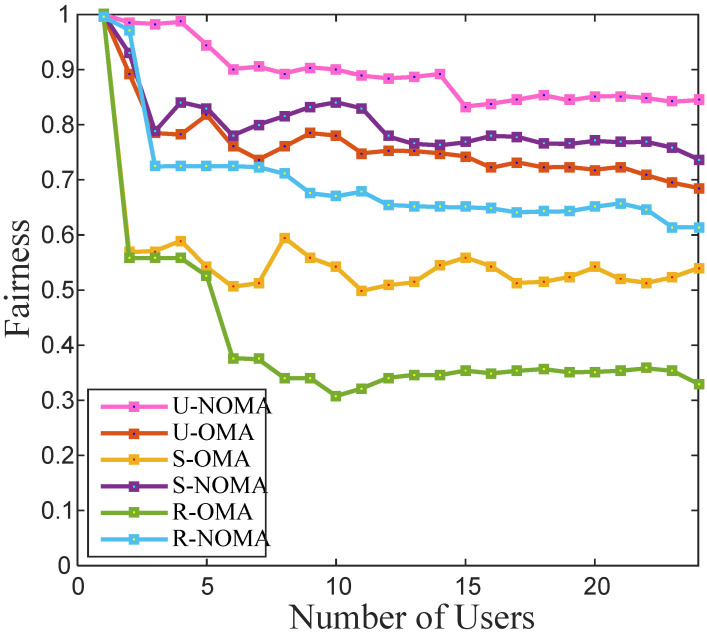
Fairness factor of various schemes for a different number of total users in the cell.

The fairness factor for N number of users lies between $\frac{1}{N}$ to 1, where 1 denotes the maximum fairness. The fairness of the system reduces with the rise in the number of users. The fairness factor defines resource allocation fairness, which describes the quality of the signal available to the users with poor channel qualities. The fairness factor is given in (12). Figure 3 evaluates the performance of the two schemes in the different deployment scenarios for a case of Z = 300. The figure shows that the fairness factor of the NOMA scheme is better than the OMA scheme. As the channel gain is poorest in the case of OMA in the rural scenarios, its fairness factor is the poorest, as observed from the figure.

**Figure 4:**
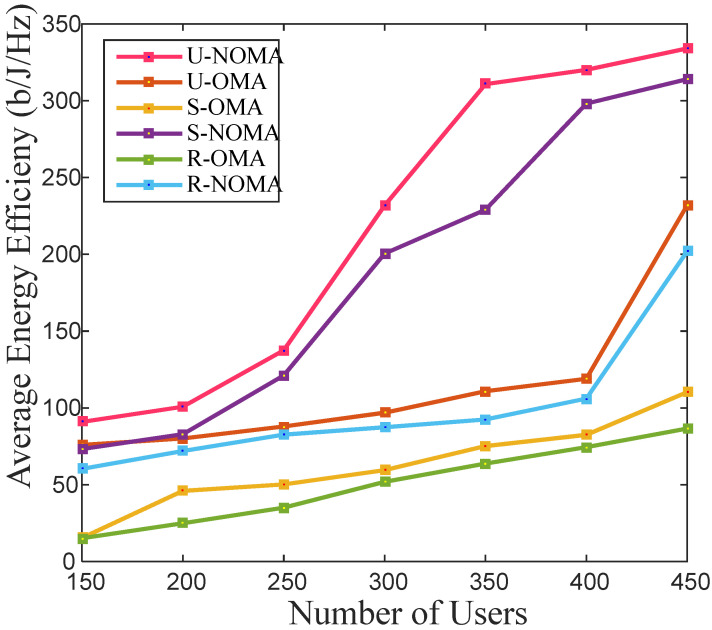
Average Energy Efficiency for a different number of total users in the cell.

For 5G, power-saving and energy optimization in the communication system are crucial. The energy efficiency of the link between the BS and cell-edge user in a coordinated relay OMA and NOMA system is shown in Figure 4. It is found that the average energy efficiency takes an upswing with the rise in the cell users. Energy efficiency is a function of achieved data rate and equipment power consumption. It is observed from Figure 2 that the rate in the rural scenario is the poorest; therefore, its energy efficiency is also the least, as seen in Figure 4. The NOMA scheme serves multiple users simultaneously, whereas OMA can use servers one user at a time. Therefore, the NOMA scheme outstrips the OMA scheme in terms of average energy efficiency.

## 6. Conclusions

This paper analyzes cooperative relaying in both OMA and NOMA techniques in a system where the relay nodes aid the communications between the BS and the cell-edge users. A practical scenario of non-uniform relay battery power levels is considered. The performance of the two schemes is compared in a multi-user system in three different deployment scenarios. It is proved that the NOMA scheme outstrips the OMA scheme in terms of average sum rate and average energy efficiency. Furthermore, it is shown that the NOMA scheme provides better average fairness to the system.

Performance analyses of OMA and NOMA systems in a heterogeneous network can be a promising future research direction in 5G. Further, with the introduction of the Internet of Things (IoT) and Device-to-Device (D2D) communications, security is a big issue in the 5G communication system. Implementation of cooperative-NOMA with physical layer security is another 5G future research direction.

## Figures and Tables

**Table 1:** Comparative analysis of this paper with existing works.

Ref. No.	Algorithm Description	Parameters Optimized	Scenario	Type of Network
[13]	To study OMA, cooperative NOMA, and NOMA schemes and propose a scheme that maintains QoS for both near and far users.	Spectral Efficiency, Sum Rate, Energy Efficiency	Downlink	Two users
[14]	According to CSI and the state of the buffer designed, the transmit power at each user to control inter-user interference and switch between the NOMA and OMA	Throughput, Outage probability, Delay	Uplink	Two users
[15]	To enhance the sum rate by OMA and NOMA according to relay serving capabilities. Propose a buffer aided system to improve the outage probability.	Throughput, Outage probability, Delay	Downlink	Two users
[12]	To enhance the energy efficiency, a NOMA network with a cooperative relay system is analyzed	Energy Efficiency	Downlink	Two users
[16]	Analysis of the sum rate of MIMO-OMA and MIMO-NOMA systems	Sum rate	Downlink	Two users
[18,19]	OMA and NOMA performance evaluation in the MIMO system	Capacity	Downlink	Multi-user
[24]	OMA and NOMA latency evaluation with short-packet communications	Effective capacity	Downlink	Two-user
[25]	Joint OMA and NOMA scheme for bandwidth efficiency	Throughput, Fairness	Downlink	Multi-user
This Paper	In a practical scenario of non-uniform relay battery powers, a comparative evaluation of OMA and NOMA systems in three different deployment scenarios	Sum-rate, System Energy Efficiency and System Fairness v/s number of users	Downlink	Multi-user

**Table 2:** Notations.

Notation	Description
Z	Number of users in the cell
Ƈ	Relay users set
Ğ	Cell-edge users set
R	Number of relays
E	Total cell-edge users
Ƥ	User equipment battery power
$c_{n}$	$n^{\mathrm{th}}$ relay
$g_{m}$	$m^{\mathrm{th}}$ cell-edge user
ƥ	Transmit power of BS
${ƥ}_{max}$	Maximum BS power
${Ҏ}_{n}$	$\mathrm{Power}\;\mathrm{of}\;c_{n}$
${Ҏ}_{n\left(max\right)}$	$\mathrm{Maximum}\;\mathrm{power}\;\mathrm{of}\;c_{n}$
${ư}_{n}$	$\mathrm{BS}\;\mathrm{to}\;c_{n}$ signal
$v_{m}$	$\mathrm{Modulated}\;\mathrm{symbol}\;\mathrm{for}\;\;g_{m}$
${Ϯ}_{n}$	$\mathrm{Signal}\;\mathrm{received}\;\mathrm{at}\;c_{n}$
${\left|h_{b,n}\right|}^{2}$	$\mathrm{Channel}\;\mathrm{gain}\;\mathrm{of}\;\mathrm{BS}\;\mathrm{to}\;c_{n}$ link
${\left|h_{n,m}\right|}^{2}$	$\mathrm{Channel}\;\mathrm{gain}\;\mathrm{between}\;c_{n}$ $\mathrm{and}\;g_{m}$
$a_{m}$	$\mathrm{BS}\;\mathrm{allotted}\;\mathrm{power}\;\mathrm{coefficient}\;\mathrm{for}\;g_{m}$
$w_{m}$	$\mathrm{Relay}\;\mathrm{allotted}\;\mathrm{power}\;\mathrm{coefficient}\;\mathrm{for}\;g_{m}$
${З}_{m}$	$\mathrm{The}\;\mathrm{signal}\;\mathrm{forwarded}\;\mathrm{by}\;c_{n}$
${Ϣ}_{k}$	$\mathrm{Received}\;\mathrm{signal}\;\mathrm{at}\;g_{m}$
$SINR_{n,m}$	$\text{Signal-to-noise-ratio}\;\mathrm{at}\;g_{m}$
${Ř}_{n,m}$	$\mathrm{Data}\;\mathrm{rate}\;\mathrm{for}\;g_{m}$ $\;\mathrm{for}\;\mathrm{the}\;\mathrm{signal}\;\mathrm{transmitted}\;\mathrm{by}\;\;c_{n}$
${Ř}_{b,n}$	$\mathrm{Data}\;\mathrm{rate}\;\mathrm{for}\;c_{n}$ for the signal transmitted by BS
${Š}_{n,M}$	Sum rate of M $\;\text{cell-edge}\;\mathrm{users},\;\mathrm{for}\;\mathrm{the}\;\mathrm{signal}\;\mathrm{transmitted}\;\mathrm{by}\;\;c_{n}$
$P_{n}$	$\mathrm{Power}\;\mathrm{consumption}\;\mathrm{of}\;c_{n}$ for signal transmission to M cell-edge users
$P_{b}$	$\mathrm{BS}\;\mathrm{power}\;\mathrm{consumption}\;\mathrm{for}\;\mathrm{sending}\;a\;\mathrm{signal}\;\mathrm{to}\;\;c_{n}$
${Ȅ}_{m}$	$\mathrm{Energy}\;\mathrm{Efficiency}\;\mathrm{of}\;g_{m}$
${Ƒ}_{N}$	Fairness factor of N cell-edge users

**Table 3:** Numerical Parameters.

Parameters	Value
THz carrier frequency	0.34 THz
Bandwidth at BS employing THz channel	10 GHz
Number of users in the cell, Z	150 to 450
Cell Radius for urban scenario	500 m
Cell Radius for sub-urban scenario	1299 m
Cell Radius for rural scenario	1732 m
$\mathrm{Maximum}\;\mathrm{power}\;\mathrm{of}\;\mathrm{BS},\;\;{ƥ}_{n\left(max\right)}$	42.7 dBm
Distance between BS and relay in an urban scenario	300 to 400 m
Distance between BS and cell-edge user in the urban scenario	400 to 500 m
Distance between BS and relay in sub-urban scenario	900 to 1000 m
Distance between BS and cell-edge in sub-urban scenario	1000 to 1299 m
Distance between BS and relay in the rural scenario	1000 to 1300 m
Distance between BS and cell-edge user in the rural scenario	1300 to 1732 m
Noise Power at the receiver of relay and cell-edge user, *σ*^2^, *σ_m_*^2^	−174 dBm/Hz
$\mathrm{Range}\;\mathrm{of}\;\mathrm{power}\;\mathrm{of}\;c_{n},{Ҏ}_{n\left(max\right)}$	−40 to 10 dBm
Path loss of THz, L	$20\;log_{10}\left(\frac{4\pi }{\lambda_{c}}\right)+$ $10z\left(f\right)d\;log_{10}e$ dB [25]

## Data Availability

Not applicable.
